# The Value of Cytology in the Evaluation of Malignant Pericardial Effusions: A Systematic Review

**DOI:** 10.3390/diagnostics12020367

**Published:** 2022-02-01

**Authors:** Ranim Shartouni, Roy Shartouni, Maryam Mahmoodi, Ilias P. Nikas

**Affiliations:** School of Medicine, European University Cyprus, Nicosia 2404, Cyprus; ranimshartounimd@gmail.com (R.S.); roy.shartouni7@gmail.com (R.S.); mariya.mahmoodi@gmail.com (M.M.)

**Keywords:** pericardial fluid, sensitivity and specificity, diagnosis, prognosis, survival analysis, metastasis, pathology, cytopathology, lung neoplasms, cancer

## Abstract

Pericardial effusions can be caused by diverse etiologies, including heart-related conditions, kidney failure, trauma, infections, autoimmune diseases, and cancer. This systematic review aimed to assess the role of cytology in identifying the most prevalent cancers related to malignant pericardial effusions (MPEs), the ability of cytology, compared to histology, to detect cancer while evaluating pericardial effusions, and the prognostic impact of MPEs. Four electronic databases were investigated using a predefined algorithm, and specific inclusion and exclusion criteria. We found that the most prevalent primaries associated with MPEs were lung (especially NSCLCs), breast, hematolymphoid, and gastrointestinal cancers. MPEs tended to be hemorrhagic rather than serous or serosanguinous and to occupy larger volumes compared to non-neoplastic effusions. In addition, cytology was shown to exhibit an enhanced ability to detect cancer compared to biopsy in most of the included studies. Lastly, the presence of an MPE was associated with poor prognosis, while survival depended on the specific cancer type detected. Particularly, prognosis was found to be worse when MPEs were caused by lung or gastric cancer, rather than breast or hematolymphoid malignancies. In conclusion, evidence suggests that cytologic evaluation has a significant diagnostic and prognostic impact in patients with MPEs.

## 1. Introduction

A pericardial effusion, which is formed by the accumulation of excessive fluid within the pericardial cavity, is a prevalent manifestation clinicians face [1]. It can be caused by diverse etiologies, such as heart-related conditions (e.g., myocardial infarction or cardiac surgery), kidney failure, trauma, infections, autoimmune diseases, and cancer [1,2,3]. Cytology is one of the diagnostic modalities used to identify the cause of a pericardial effusion, particularly to detect whether cancer cells are present within the fluid [3,4]. Cancer-associated pericardial effusions might be either free from malignancy—for instance, when induced by chemotherapy or radiotherapy—or show evidence of cancer cells (malignant pericardial effusions; MPEs); the latter are most often derived from a metastasis rather than a primary lesion. Furthermore, the presence of a MPE has been associated with poor prognosis, while its management necessitates a multidisciplinary approach [3,5,6,7]. Recently, the International System for Reporting Serous Fluid Cytology (ISRSFC) has been reported with the goal to improve diagnosis, standardize reporting, and facilitate communication among physicians [8].

To our knowledge, while the cytology of MPEs has previously been described in multiple studies, no systematic review has been performed up to date. Our study aimed to highlight the diagnostic and prognostic impact of cytology while evaluating pericardial effusions.

## 2. Materials and Methods

### 2.1. Search Strategy

We performed this systematic review following the Preferred Reporting Item for Systematic Review and Meta-Analysis (PRISMA) guidelines [9]. We comprehensively investigated four electronic databases (PubMed, Embase, Scopus, and Web of Science) for articles published until May 2021 reporting on the cytopathology of MPEs. We used the following search algorithm: “pericardi* AND (cytolog* OR cytopatholog*) AND (effusion* OR fluid) AND (cancer* OR carcinoma* OR metasta*)”, while no specific search filters were applied (e.g., publication date). Duplicates were removed with the Paperpile reference manager. Then, the remaining records were inserted into the Rayyan App for title–abstract selection [10].

### 2.2. Study Selection

Two authors (Ranim Shartouni, and Ilias P. Nikas) selected independently the eligible studies using predefined inclusion and exclusion criteria (Table 1). This was done in two steps: first, an initial title-abstract screening was performed in the Rayyan App, and its results underwent a full-text evaluation to select the final eligible study list. Any disagreements were resolved by a consensus between the two authors.

### 2.3. Data Extraction

The following data were extracted from each eligible study onto an Excel^®^ spreadsheet: first author and year, country, study design, study period, follow-up duration, mean and/or median age of the enrolled patients, total number of patients and samples included, number of samples with malignant cytology and histology, malignancy rate between females and males, number of serous, hemorrhagic, serosanguinous, or purulent effusions, mean or median effusion volume of the benign, malignant or all effusions evaluated, main symptoms reported, cancer types described, and selected prognostic data (e.g., OR, overall survival; median survival; HR, hazard ratio).

### 2.4. Study Outcomes

The outcomes of this systematic review were used to assess the following:

- The role of cytology in identifying specific cancer primaries associated with MPEs;
- The ability of cytology, compared to histology, to detect cancer while evaluating pericardial effusions;
- The prognostic impact of MPEs.

## 3. Results

### 3.1. Literature Search

**Figure 1:**
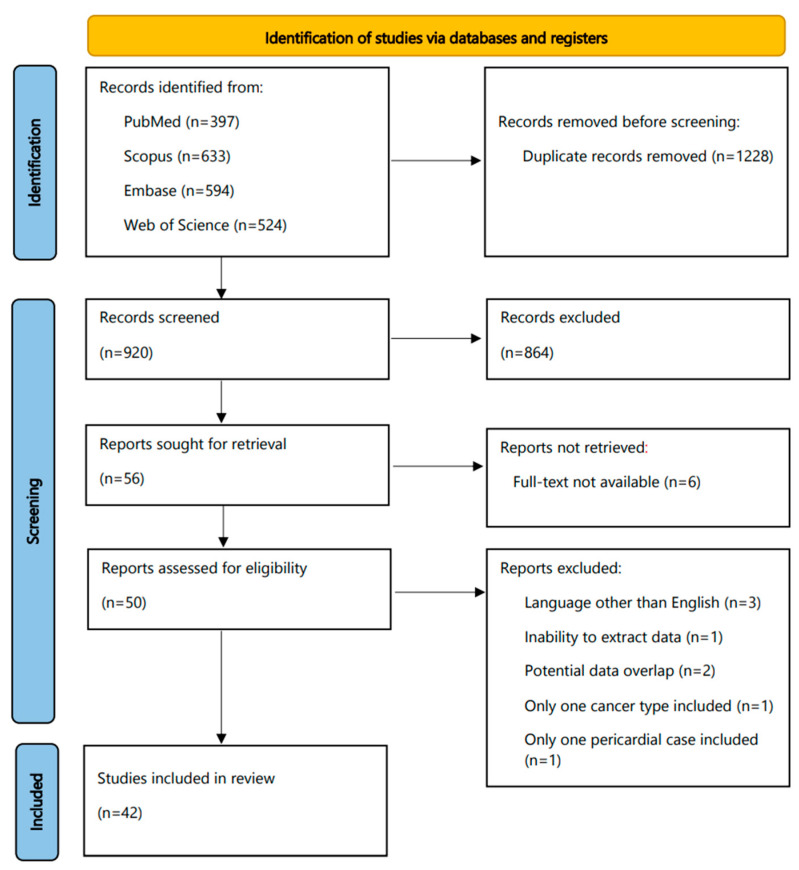
Flowchart of this systematic review.

The flowchart of our study is displayed in Figure 1. The search yielded initially a total number of 2148 articles (PubMed, 397; Scopus, 633; Embase, 594; Web of Science, 524); of them, 1228 were removed as duplicates. Then, 920 reports were screened in a title–abstract fashion. The whole process resulted in 42 studies included in our review for subsequent data extraction and analysis.

### 3.2. Study and Patient Characteristics

Table S1 displays the characteristics of the 42 eligible studies, conducted from 1972 to 2020 across various countries, most often (*n* = 21) in the U.S.A. The majority of them had a retrospective design. Table 2 shows the percentage of pericardial malignancy in females vs. males, also highlights the percentage of samples where cancer was detected. A total number of 9570 patients was included (4490 females and 5080 males), while a total number of 9156 samples was also recorded, of which 2807 were positive for malignancy. In seven studies [3,4,11,12,13,14,15], females had a higher malignancy prevalence, ranging from 57% to 82%; in contrast, MPEs were more prevalent in males in three studies [16,17,18], ranging from 70.7% to 72.3%. MPEs tended to be hemorrhagic [12,16,19,20,21,22], rather than serous or serosanguinous, and also to occupy larger volumes than benign effusions [13,14,15,19,23] (Table S2).

### 3.3. Cancer Types Associated with MPEs

Table S3 shows the most prevalent cancer primaries in the patients with cancer-associated pericardial effusions (effusions in patients with clinical history of cancer, associated with presence or absence of malignant cells) included in each eligible report. Lung cancer was the most common primary in most studies, followed by breast cancer, hematolymphoid, and gastrointestinal malignancies. Other primaries comprised gynecological cancers, thyroid and urinary malignancies, melanomas, sarcomas, germ cell tumors, and thymomas. Concerning mesotheliomas, these occupied only a minority of the cases extracted. We found 18 cases in total, of which 6 were reported as pleural mesotheliomas. In the two studies focusing on pediatric MPEs, hematolymphoid neoplasms and sarcomas were the ones most often reported [37,39].

**Figure 2:**
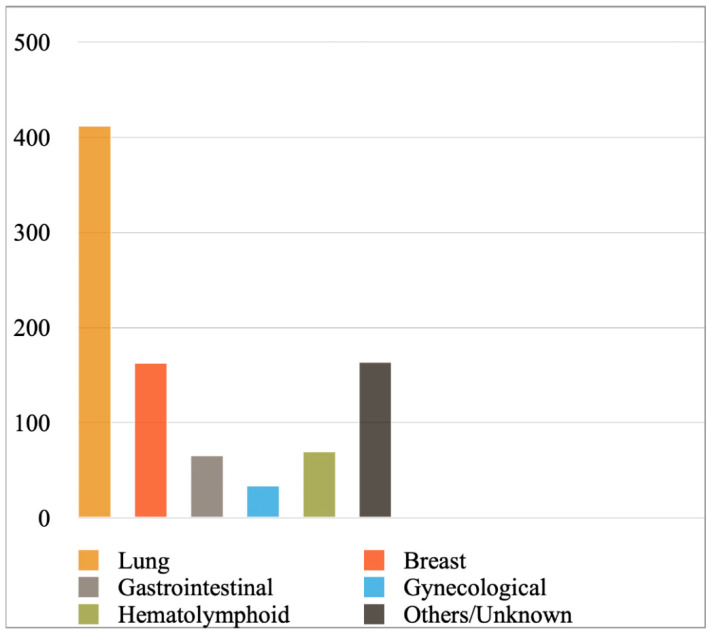
Most prevalent cancer primaries in malignant pericardial effusions. In the included studies, results were reported with cytology or a combination of cytology and biopsy.

**Figure 3:**
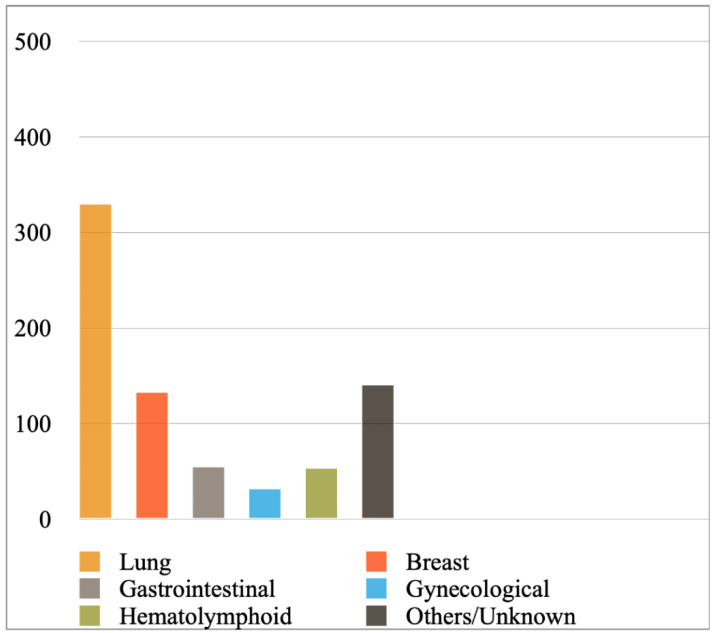
Most prevalent cancer primaries in malignant pericardial effusions diagnosed exclusively with cytology.

Figure 2 shows the most prevalent cancer primaries in MPEs diagnosed with cytology or a combination of cytology and biopsy in the included studies, while Figure 3 shows their prevalence when results were reported exclusively with cytology. Lung cancer (comprising mainly adenocarcinomas) was reported to be the most common primary, followed by breast cancer, hematolymphoid and gastrointestinal malignancies. Briefly, from the studies reporting findings derived either from cytology or a combination of cytology and biopsy (Figure 2), we calculated the following frequencies: lung cancer 45%, breast cancer 18%, hematolymphoid neoplasms 8%, gastrointestinal cancers 7%, gynecological cancers 4%, and others/unknown 18%. In this analysis, the study by Wilkes et al. [46] was excluded, as it did not report separately the numbers of gastrointestinal and gynecological cancer diagnoses. In addition, from the studies reporting only cytologic findings (Figure 3), we calculated the following frequencies: lung cancer 44%, breast cancer 18%, hematolymphoid neoplasms 7%, gastrointestinal cancers 7%, gynecological cancers 4%, and others/unknown 19%. Analytical results of the reported cancer types in each eligible study are shown in Tables S4 and S5, respectively. Concerning lung cancer, non-small cell lung carcinoma (NSCLC) was much more common than small cell lung carcinoma (SCLC); in the MPEs diagnosed with cytology only, just 13 cases were confirmed as SCLC (out of 330 lung cancer cases in total; Table S5).

### 3.4. Cytology vs. Histology for Cancer Detection While Evaluating Pericardial Effusions

Table 3 compares the performance of cytology and histology for diagnosing MPEs in the studies that applied both modalities. For this analysis, and wherever present, cytologically suspicious interpretations were regarded as positive, and atypical or indeterminate interpretations as negative. Cytology tended to be positive for malignancy in cases where histology was negative [3,11,12,13,16,23,38,40,43,44,46] more often than the opposite scenario [15,34,48,49]. Although in most of these studies the difference was not statistically significant (Table 3), the following three studies exhibited a significantly higher ability of cytology to detect cancer in the pericardium, compared to biopsy. First, in the report by Maisch et al. [16], 32 cases were found to be positive for malignancy only with cytology, as their paired biopsies yielded negative results; meanwhile, histology samples detected just five cancers missed with cytology. Thus, cytology detected more cancers involving the pericardium than histology (*p* < 0.001). Likewise, statistically significant results were reported by Cullinane et al. [24] and Lopez et al. [12] (*p* = 0.02 and *p* = 0.04, respectively), both highlighting the value of cytology detecting MPEs. In contrast, in none of the studies where histology detected more cancers than cytology [15,34,48,49] was the result statistically significant.

Notably, the combination of both cytology and biopsy could increase the overall diagnostic performance. For instance, Wilkes et al. reported sensitivities of 90% and 56% with cytology and histology, respectively, while their combination increased the overall sensitivity to 94% [46].

Three studies reported their pericardial effusion findings using the newly developed ISRSFC [11,14,30], stratifying their interpretations into each of the five reporting categories linked with a different risk of malignancy (ROM). Lobo et al. reclassified 64 samples using the criteria of ISRSFC and reported a 100% risk of malignancy (ROM) for the malignant category; in contrast, the negative and atypical categories exhibited a 0% ROM.

### 3.5. Prognostic Impact of MPEs

The presence of MPEs, diagnosed either with cytology or biopsy, was correlated with dismal prognosis [6,13,20,22,24,26,31,33,38]. Notably, the specific cancer primary seems to be important. Cullinane et al. reported that lung cancer was associated with shorter survival than breast or hematolymphoid malignancies [24], while Gornik et al. reported that patients with breast and hematolymphoid cancers exhibited longer survival compared to lung and other solid malignancies [26]. Kil et al. also displayed that patients with breast cancer or lymphoma showed longer survival than stomach cancer or unknown metastases [33], while He et al. reported that cases with lung cancer exhibited worse prognosis than non-cancer cases [22]. Lekhakul et al. reported that a diagnosis of chronic myelogenous leukemia or lymphoma was linked with a better prognosis compared to carcinoma or sarcoma [35]. Lastly, Wilkes et al. studied a cohort largely composed of malignant effusions from hematopoietic neoplasms and chemo/radiosensitive breast cancers; of interest, survival did not statistically differ from non-malignant effusions [46]. A summary of the relevant prognostic findings in the included studies is shown in Table 4.

## 4. Discussion

To our knowledge, this is the first systematic review examining the role of cytology in the evaluation of MPEs. We found that the most prevalent malignancies detected in MPEs, either with cytology or biopsy, were derived from lung, breast, hematopoietic, or gastrointestinal primaries. In addition, most studies showed that cytology exhibited an enhanced ability to detect cancer compared to biopsy. Lastly, the presence of a MPE was associated with dismal prognosis, while survival depended on the specific cancer type detected.

Similar to our study that focused on pericardial effusions, the prevalence of various cancers has also been shown in pleural and peritoneal effusions. Dermawan et al. reported that the most common malignancies associated with malignant pleural effusions in males were derived from lung (especially adenocarcinoma), hematolymphoid, genitourinary, and gastrointestinal primaries, whereas they were derived from breast, Mullerian, lung, and hematolymphoid primaries in females. They also found that hematolymphoid, gastrointestinal, and genitourinary malignancies were the most common ones associated with malignant peritoneal effusions in males; in contrast, primaries of Mullerian origin, the breast, and the gastrointestinal system were most often found in females [4]. Of interest, Flanagan et al. investigated the Irish National Cancer Registry for extra-abdominal cancers metastasizing to the peritoneum, reporting that the most common primaries were the breast cancer, lung cancer, and melanoma. In addition, peritoneal metastasis was associated with poor prognosis compared to stage IV metastatic cancers devoid of peritoneal cancer spread [50].

Our study additionally reported that MPEs were more likely to be hemorrhagic and occupy larger volumes than non-malignant effusions. Likewise, the presence of hemorrhage has also been associated with malignant pleural effusions [51]. A potential cause could be the high levels of VEGF secreted by the cancer cells [52]. Notably, a recent study compared malignant pleural effusions with and without hemorrhage and found that the former was associated with more severe dyspnea and larger effusion volumes, in addition to worse prognosis and response to therapy [53].

According to our results, cytology tended to be positive for malignancy in cases where histology was negative [3,11,12,13,16,23,38,40,43,44,46] more often than the opposite scenario [15,34,48,49], potentially exhibiting an enhanced cancer detection ability while evaluating the pericardium. The lower performance of histology could be because of sampling error, as the pericardium is more difficult to biopsy than the pleura or peritoneum [18]. Other reasons could be the fact that cancer cells first spread to the visceral, before reaching the parietal, pericardium, in addition to the reported dispersed involvement of the pericardium in metastatic disease [46]. Notably, results from a recent study suggest another potential explanation for the enhanced ability of cytology to detect MPEs. Karpathiou et al. showed that pericardial metastases often exhibit a pattern where cells float inside the cavity. In contrast, pleural metastases tend to present with an invasive pattern [54]. Consequently, cytologic sampling seems a suitable modality to detect cancers involving the pericardium, whereas tissue biopsy could be more likely to be successful when evaluating pleural metastases. However, the combination of both cytology and tissue biopsy may increase the overall diagnostic accuracy while evaluating MPEs [46]. Of interest, the application of the recent ISRSFC could further enhance cytology’s diagnostic performance, facilitate communication, and minimize interobserver variability [11,14,30,55,56]; however, more studies are needed to unravel its potential.

Our review highlighted that MPEs were linked with higher mortality rates, while positive pericardial cytology was a significantly dismal prognostic factor [6,13,20,22,24,26,31,33,38]. Notably, the specific cancer primary seemed to be important, as lung cancer and gastric cancer were associated with worse prognosis than breast or hematolymphoid malignancies [24,26,33]. Of interest, a recent study showed that survival was shorter for patients with MPEs associated with gastric cancer, compared to lung, breast, or other malignancies [56]. Prognosis has been reported to be similarly poor in patients with malignant pleural or peritoneal effusions [57,58]. In a study enrolling patients with malignant pleural effusions, lung and gastric cancers were found to be worse prognostic factors compared to breast, ovarian, or renal carcinomas [57].

This systematic review has some important limitations. It was composed mainly of retrospective studies, while most were of a small size. In addition, many of the included studies exhibited possible selection bias, while their results were largely reported in a heterogenous way. Furthermore, a cytohistological correlation could not be performed in all studies, let alone in all samples in each study individually. In five of the studies included in the cytology/histology correlation analysis (Table 3), cytology interpretations originally reported as suspicious or positive were both considered as being cytologically positive for this analysis; this could have potentially overestimated the ability of cytology to detect cancer in these particular studies. Lastly, the heterogeneity in the reporting of prognostic findings (various outcomes) prohibited their pooled analysis.

## 5. Conclusions

This study showed that the most prevalent primaries associated with MPEs are lung (especially NSCLCs), breast, hematolymphoid, and gastrointestinal cancers. MPEs tend to be hemorrhagic rather than serous or serosanguinous and occupy larger volumes than non-neoplastic effusions. Notably, cytology may exhibit enhanced cancer detection ability while assessing pericardial effusions, compared to histology. Lastly, MPEs show poor prognosis, while evidence suggests that the latter is worse when MPEs are caused by lung or gastric cancer, rather than breast or hematolymphoid malignancies.

## Figures and Tables

**Table 1:** Inclusion and exclusion criteria of this systematic review.

**Inclusion Criteria**
Malignant pericardial effusions diagnosed with cytology (with or without histology correlation)
Testing on humans
**Exclusion Criteria**
Articles without any pericardial effusion data (e.g., containing only pleural, peritoneal fluid data)
Articles describing only non-neoplastic pericardial effusions
Articles without any cytologic data (e.g., containing only tissue biopsy data)
Testing on animal models or cell lines only
Case reports, conference abstracts, reviews, and editorials
Small case series (less than or equal to five patients)
Languages other than English
Articles describing only a single cancer type (e.g., mesothelioma)
Articles describing only a single category of cancers (e.g., lymphoid neoplasms)
Inability to extract data

**Table 2:** Presence of pericardial malignancy in the included studies.

First Author, Year	Total No. of Patients	Gender (F/M)	% Malignancy (F/M)	Total No. of Samples	No. of Samples with Malignant Cytology	% Malignancy (Samples)
Bardales et al., 1996 [18]	96	33/63	12/29 (29.3%/70.7%)	112	45	40.18%
Campbell et al., 1992 [13]	25	15/10	15/10 (60%/40%)	25	11	44.00%
Cullinane et al., 2004 [24]	63	41/22	NA	58	28	48.28%
Dermawan and Policarpio-Nicolas, 2020 [4]	1285	658/627	88/67 (57%/43%)	1285	155	12.06%
Di Liso et al., 2019 [25]	29	14/15	NA	15	10	66.67%
Dragoescu and Liu, 2013 [3]	113	57/56	23/8 (74.2%/25.8%)	128	31	24.22%
Edoute et al., 1992 [19]	62	21/21	21/21 (50%/50%)	60	42	70.00%
García-Riego et al., 2001 [17]	375	18/47	18/47 (27.7%/72.3%)	375	65	17.33%
Gecmen et al., 2017 [21]	283	121/162	NA	283	44	15.55%
Gornik et al., 2005 [26]	219	103/116	NA	182	52	28.57%
Gupta et al., 2000 [27]	76	30/46	NA	76	22	28.95%
Gupta et al., 2012 [28]	NA	NA	NA	204	10	4.90%
Haskell and French, 1985 [29]	56	NA	NA	27	22	81.48%
He et al., 2017 [22]	116	38/78	NA	116	43	37.07%
Hou et al., 2020 [30]	NA	NA	NA	2405	1260	52.39%
Jeon et al., 2014 [31]	55	24/31	NA	55	34	61.81%
Kabukcu et al., 2004 [32]	50	16/34	NA	50	15	30.00%
Kil et al., 2007 [33]	116	65/51	NA	116	27	23.28%
Krikorian and Hancock, 1978 [34]	123	65/58	NA	96	16	16.70%
Lekhakul et al., 2018 [35]	171	80/91	NA	164	95	58.00%
Lobo et al., 2020 [11]	56	43/21	27/13 (68%/32%)	64	40	62.50%
Lopez et al., 1983 [12]	12	8/4	8/4 (67%/33%)	12	11	91.60%
Maisch et al., 2010 [16]	68	28/40	12/30 (28.6%/71.4%)	68	42	61.76%
Malamou-Mitsi et al., 1995 [36]	44	23/ 21	NA	53	16	36.36%
Medary et al., 1996 [37]	9	2/7	NA	9	1	11.11%
Mirhosseini et al., 2012 [38]	153	64/89	NA	113	50	44.25%
Neragi-Miandoab et al., 2008 [6]	62	28/34	NA	48	27	56.25%
Parsons and Jarzembowski, 2016 [39]	NA	NA	NA	28	3	10.70%
Patel et al., 2013 [40]	88	53/35	NA	88	43	48.86%
Razek and Samir, 2019 [41]	41	12/29	NA	28	17	42.50%
Robles et al., 1997 [15]	22	13/9	5/3 (62.5%/37.5%)	22	4	18.18%
Rodriguez et al., 2020 [14]	299	162/137	28/6 (82%/18%)	299	34	11.37%
Rossi et al., 2015 [42]	3171	1463/1708	NA	252	36	14.29%
Saab et al., 2016 [43]	364	188/176	NA	419	62	15.00%
Sarigul et al., 1999 [23]	305	107/198	NA	38	14	36.80%
Strobbe et al., 2017 [20]	269	119/150	NA	208	68	32.69%
Volk et al., 2019 [44]	113	56/57	NA	113	16	14.16%
Wagner et al., 2010 [45]	174	114/65	NA	179	NA	NA
Wilkes et al., 1995 [46]	127	63/64	NA	112	65	58.04%
Yonemori et al., 2007 [47]	88	30/30	NA	88	60	68.18%
Zhu et al., 2015 [48]	1022	550/472	NA	1022	158	15.46%
Zipf and Johnston, 1972 [49]	47	NA	NA	61	13	27.66%

Abbreviations: NA, not available; F, female; M, male.

**Table 3:** Comparison of cytology and histology for detecting cancer in pericardial effusions. The studies showing differences in their cytology vs. histology findings are compared, and the resulting *p*-values are shown in the Table. Results exhibiting differences between cytology and histology, also *p*-values < 0.05, are highlighted with **Bold**.

First Author, Year	C (+)/H (+)	C (+)/H (−)	C (−)/H (+)	C (−)/H (−)	*p*-Value	C (+)/H (NA)	C (+)/H (NA)
Bardales et al., 1996 [18]	45	0	0	16		0	51
Campbell et al., 1992 [13]	5	**6**	**0**	14	0.08	0	0
Cullinane et al., 2004 [24]	15	**13**	**0**	28	**0.02**	0	2
Dragoescu and Liu, 2013 [3]	6	**6**	**3**	30	0.62	19	64
‡ Edoute et al., 1992 [19]	7	2	2	1		35	13
He et al., 2017 [22]	13	0	0	0		30	73
Jeon et al., 2014 [31]	34	0	0	21		0	0
Kabukcu et al., 2004 [32]	1	0	0	0		14	35
Krikorian and Hancock, 1978 [34]	16	**0**	**2**	7	0.76	0	39
‡ Lobo et al., 2020 [11]	10	0	0	3		30	21
Lopez et al., 1983 [12]	5	**6**	**1**	0	**0.04**	0	0
Maisch et al., 2010 [16]	5	**32**	**5**	26	**<0.001**	0	0
‡ Malamou-Mitsi et al., 1995 [36]	10	0	0	9		6	19
Mirhosseini et al., 2012 [38]	30	**20**	**8**	55	0.13	0	0
Patel et al., 2013 [40]	15	**9**	**2**	13	0.17	19	30
Robles et al., 1997 [15]	0	**0**	**2**	16	0.49	4	0
‡ Rossi et al., 2015 [42]	36	0	0	30		0	186
Saab et al., 2016 [43]	18	**17**	**5**	142	0.11	27	210
Sarigul et al., 1999 [23]	8	**5**	**4**	19	1.00	1	1
Strobbe et al., 2017 [20]	4	1	1	8		63	131
Volk et al., 2019 [44]	8	**8**	**2**	95	0.30	0	0
‡ Wilkes et al., 1995 [46]	34	**13**	**3**	23	0.13	18	21
Zhu et al., 2015 [48]	15	**0**	**6**	18	0.18	143	838
Zipf and Johnston, 1972 [49]	13	**0**	**2**	32	0.82	0	0

Note: The Fisher’s exact test was used to compare the ability of cytology vs. biopsy to detect cancer while evaluating pericardial effusions; *p*-values < 0.05 were considered significant. ‡ In these studies, positive cytology (C+) included both suspicious and positive original interpretations. Abbreviations: NA, not available; C, cytology; H, histology.

**Table 4:** Studies highlighting the prognostic impact of cancer-associated and malignant pericardial effusions.

First Author, Year	Main Prognostic Findings
Campbell et al., 1992 [13]	In a cohort composed of 25 patients with malignancy, a higher 12-month mortality was found in MPEs compared to noncancerous effusions (91% vs. 57%, respectively)
Cullinane et al., 2004 [24]	When evaluated preoperatively, the presence of a MPE confirmed either with cytology or biopsy and a NSCLC diagnosis were associated with shorter OS (*p* = 0.02, *p* = 0.02, and *p* = 0.0014, respectively) Effusions associated with lung cancer were associated with shorter survival rate than breast (*p* = 0.02), other solid cancers (*p* = 0.02), or hematolymphoid malignancies (*p* = 0.05)
Gornik et al., 2005 [26]	In a cohort composed of cancer-associated pericardial effusions, the presence of suspicious/malignant cytology was linked with shorter median survival compared to normal cytology (7.3 vs. 29.7 weeks; *p* = 0.02) When analyzing the subgroup composed of lung cancer-associated pericardial effusions, suspicious/positive cytology was linked with a shorter median survival compared to normal cytology (6.1 vs. 40.4 weeks, respectively, *p* = 0.0139) Effusions associated with lung or other solid cancers were associated with a reduced median survival (11.1 and 5.1 weeks, respectively) in comparison to hematolymphoid malignancies or breast cancer (45.6 and 43.0 weeks, respectively, log-rank *p* = 0.0012)
He et al., 2017 [22]	MPEs showed shorter median OS compared to the non-malignant ones (4 months vs. 10 months, respectively; *p* < 0.05)
Jeon et al., 2014 [31]	Patients with MPEs, confirmed either with cytology or biopsy, exhibited decreased OS (HR = 1.964; 95% CI, 1.053–3.663; *p* = 0.034) The median survival was 2 months for the positive, yet 8 months for the negative pericardial effusions
Kil et al., 2008 [33]	MPEs were associated with a higher 6-month mortality rate than the non-malignant ones (80.3% vs. 18.2%); patients with lung cancer exhibited the highest mortality (84.4%) Patients with MPEs showed shorter survival than the ones with normal effusions (log-rank; *p* < 0.0001) Patients with lymphoma or breast malignancies exhibited longer survival (11.4 and 7.7 months, respectively), than patients with stomach malignancies or metastases of unknown origin (1.2 and 2.3 months, respectively)
Lekhakul et al., 2018 [35]	Patients with hematolymphoid malignancies exhibited longer survival compared to patients with either sarcomas or carcinomas (median survival: 102 vs. 12 weeks, *p* < 0.0001; 5-year survival: 46% vs. 3%, *p* < 0.0001) Patients with hematolymphoid malignancies exhibited lower mortality than the ones from sarcomas or carcinomas (*p* < 0.001)
Mirhosseini et al., 2012 [38]	History of positive lung (HR = 2.894; 95% CI, 1.362–6.147; *p* = 0.006) or other organ malignancy (HR: 2.315; 95% CI, 1.009–50311; *p* = 0.048) were independent predictors of death following surgery Positive cytology was associated with shorter OS (*p* = 0.001)
Neragi-Miandoab et al., 2008 [6]	Mean survival was shorter in patients with positive pericardial cytology compared to patients with negative cytology (4.89 +/− 0.9 months vs. 13.4 +/− 0.98 months, respectively; *p* = 0.0175) Effusions associated with esophageal and lung cancer exhibited shorter 2-year OS, compared to “other cancers” (17.8% vs. 32%, respectively; *p* = 0.06); in the 5-year survival analysis, no patient with esophageal or lung cancer survived after 46 months, whereas the survival rate was 16.2% in the “other cancers” group
Strobbe et al., 2017 [20]	Cancer-associated pericardial effusions were associated with shorter survival (HR = 3.01; 95% CI, 1.66–5.45; *p* < 0.001) MPEs were associated with shorter survival compared to the non-malignant ones (HR = 3.31; 95% CI, 2.37–4.61; *p* < 0.001)
Wilkes et al., 1995 [46]	In a cohort composed of 127 cancer-associated pericardial effusions, most patients that survived 12 months or more had a hematolymphoid malignancy or breast cancer
Yonemori et al., 2007 [47]	Positive pericardial cytology was associated with shorter OS (HR = 3.1; 95% CI, 1.5–6.3; *p* = 0.001)

Abbreviations: MPEs, malignant pericardial effusions; NSCLC, non-small cell lung carcinoma; OS, overall survival; HR, hazard ratio.

## Data Availability

Data are contained within the article or Supplementary Material.

## Supplementary Materials

The following supporting information can be downloaded at: https://www.mdpi.com/article/10.3390/diagnostics12020367/s1, Table S1: Study Characteristics; Table S2: Gross findings of pericardial effusions and their association with malignancy; Table S3: Most prevalent primary cancer sites/neoplasms in patients presenting with cancer-associated pericardial effusions; Table S4: Most prevalent primary cancer sites/neoplasms in patients presenting with malignant pericardial effusions diagnosed with cytology and/or biopsy; Table S5: Most prevalent primary cancer sites/neoplasms in patients presenting with malignant pericardial effusions diagnosed exclusively with cytology.
