# Supplementary material for: The Value of Cytology in the Evaluation of Malignant Pericardial Effusions: A Systematic Review
Source: Diagnostics (Basel). 2022 Feb 1;12(2):367. doi: 10.3390/diagnostics12020367 (PMC8871495; doi:10.3390/diagnostics12020367)
Supplement: Supplementary file 1 [file diagnostics-12-00367-s001.zip › Pericardial SR Supplemental File.pdf]

Table S1. Study Characteristics

First Author, Year	Country	Study Design	Study Period	Follow up Duration	Age Mean (SD)	Age Median (IQR)	Age Range (years)
Lobo et al., 2020	Portugal	Retrospective	2008 - 2018	NA	NA	NA	9 - 79
Lopez et al., 1983	Venezuela	Retrospective	1979 - 1982	NA	39.8 (12.80)	44	18 - 55
Razek & Samir, 2019	Egypt	Retrospective	2006 - 2016	NA	39 (NA)	NA	22 - 68
Patel et al., 2013	USA	Retrospective	2000 - 2007	1 year	60 (14)	NA	NA
Sarigul et al., 1999	Turkey	Retrospective	1984 - 1996	2 - 54 months	26.98 (11.12)	NA	15 days - 75 years
Wilkes et al., 1995	USA	Retrospective	1968 - 1994	57 days - 15 years	NA	50	17 - 77
Edoute et al., 1992	Israel	Retrospective	1977 - 1989	NA	NA	56	13 - 79
Campbell et al., 1992	USA	Prospective	1987 - 1988	>12 months	52.64 (10.88)	53	26 - 70
Krikorian & Hancock, 1978	USA	Retrospective	1970 - 1976	NA	NA	46	6 - 80
Hou et al., 2020	USA	Retrospective	Jan - Dec 2014	NA	NA	NA	NA
Rodriguez et al., 2020	Brazil & USA	Retrospective	2014 - 2019	8 months	51.25 (NA)	NA	<1 - 89
Dermawan & Policarpio-Nicolas, 2020	USA	Retrospective	2000 - 2016	NA	NA	65	3 - 97
Volk et al., 2019	USA	Retrospective	2011 - 2018	NA	59.7 (15.1)	NA	NA
Lekhakul et al., 2018	USA	Prospective	2007 - 2016	<5 years	NA	60 (15)	NA
Strobbe et al., 2017	Belgium	Retrospective	2006 - 2016	10 years	62 (15.9)	NA	0 - 94
Gecmen et al., 2017	Turkey	Retrospective	2007 - 2016	NA	60 (16.6)	NA	NA
Saab et al., 2016	USA	Retrospective	2008 -	<190 days	63 (NA)	NA	2 - 95

			2014				
He et al., 2017	China	Retrospective	2012 - 2016	1 month - 43 months	56.4 (NA)	NA	13 - 83
Rossi et al., 2015	Brazil, Italy, Luxembourg, Portugal	Prospective	2000 - 2013	NA	NA	48	23 - 92
Zhu et al., 2015	USA	Retrospective	1983 - 2013	NA	56 (NA)	NA	8 days - 85 years
Jeon et al., 2014	Republic of Korea	Retrospective	2003 - 2012	NA	57 (NA)	57	29 - 82
Dragoescu & Liu, 2013	USA	Retrospective	2005 - 2010	NA	52.6 (NA)	NA	6 - 85
Gupta et al., 2012	India	Retrospective	1998 - 2007	NA	51.5 (NA)	NA	6 - 85
Mirhosseini et al., 2012	Iran	Retrospective	2004 - 2011	Median: 15 months	50.3 (15.5)	NA	14 - 84
Wagner et al., 2010	USA	Retrospective	2004 - 2009	NA	NA	57.4	1 - 86
Maisch et al., 2010	Germany & Serbia	Observational	1988 - 2008	<1 year	58.8 (13.2)	NA	NA
Hyun Kil et al., 2007	Republic of Korea	Retrospective	1995 - 2007	Mean: 28 months	56 (39)	NA	NA
Neragi-Miandoab et al., 2008	USA	Retrospective	1999 - 2004	NA	58.4 (14.3)	NA	19 - 79
Yonemori et al., 2007	Japan	Retrospective	1998 - 2005	NA	NA	58	32 - 72
Gornik et al., 2005	USA	Retrospective	1999 - 2003	1 year	57.5 (1)	NA	20.8 - 93.5
Cullinane et al., 2004	USA	Retrospective	1990 - 2001	<8.3 months	50	NA	20 - 82
García-Riego et al., 2001	Spain	Retrospective	1976 - 1999	NA	53.6 (NA)	NA	4 - 77
Gupta et al., 2000	New Zealand	Retrospective	1991 - 1998	NA	52/F - 59/M	NA	NA
Robles et al., 1997	Spain	Retrospective	1992 - 1995	Mean: 20.5 months	52.4 (NA)	54	18 - 81

Malamou-Mitsi et al., 1995	Greece	Retrospective	>7 years	NA	56.9 (NA)	NA	16 - 91
Bardales et al., 1996	USA	Retrospective	1984 - 1993	NA	NA	57	14 - 82
Haskell & French, 1985	USA	Retrospective	1977 - 1984	NA	NA	NA	NA
Zipf & Johnston, 1972	USA	Retrospective	NA	NA	NA	NA	NA
Kabukcu et al., 2004	Turkey	Retrospective	1998 - 2002	Median: 18 months	51 (17)	NA	12 - 80
Liso et al., 2019	Italy	Retrospective	2011 - 2017	NA	NA	62	39 - 81
Parsons & Jarzembowski, 2016	USA	Retrospective	2000 - 2015	Mean: 4.7 years	NA	NA	NA
Medary et al., 1996	USA	Retrospective	1984-1995	4 months - 7 years	NA	14 years	4 months-7 years

Abbreviations: NA, information not available

Table S2. Gross findings of pericardial effusions and their association with malignancy.

First Author, Year	No of Serous Effusions (Malignant)	No of Hemorrhagic Effusions (Malignant)	No of Purulent Effusions (Malignant)	No of Serosanguinous Effusions (Malignant)	Benign/Total Effusion Volume	Malignant Effusion Volume
Lopez et al., 1983	0	12 (12)	0	0	NA / 400 - 1000 mL	400 - 1000 mL
Patel et al., 2013	NA	NA	NA	NA	NA	mean: 814 mL
Sarigul et al., 1999	NA	NA	NA	NA	median: 975.25 ± 48.46 mL / NA	median: 1131.25 ± 97.48 mL
Wilkes et al., 1995	0	0	0	0	mean: NA / 509 mL (range 5 - 2300 mL)	NA
Edoute et al., 1992	7 (3)	48 (38)	2 (0)	0	median: 470 mL (range 70 - 1750) / NA	median: 630 mL (range 80 - 2250)
Campbell et al., 1992	0	0	0	0	mean: 434 mL ± 357 mL / (range 100 - 1250)	mean: 554 mL ± 332

Rodriguez et al., 2020	0	0	0	0	mean: 300.3 mL (range 3 - 1700) / mean: 299 mL (range 3 - 1700)	mean: 307 mL (range 8 - 800)
Lekhakul et al., 2018	53 (NA)	35 (NA)	0	83 (NA)	NA / median: 485 mL (IQR 350-719)	NA
Strobbe et al., 2017	62 (14)	149 (38)	0	53 (16)	NA	NA
Gecmen et al., 2017	108 (2)	175 (42)	0	0	NA	NA
Saab et al., 2016	0	0	0	0	mean: 75 mL (range, 10-900 mL) / NA	mean: 49 mL (range, 25-900 mL)
He et al., 2017	0	NA (43)	0	0	NA	NA
Jeon et al., 2014	18 (NA)	0	0	37 (NA)	NA / median: 500 mL (range 100 to 1500 mL)	NA
Dragoescu & Liu, 2013	0	0	0	0	NA / mean: 61 mL (range < 1 mL to 1150 mL)	NA
Mirhosseini et al., 2012	53 (NA)	69 (NA)	0	0	NA / mean: 752 ± 429 mL	NA
Maisch et al., 2010	0	25 (23)	0	0	NA	NA
Hyun Kil et al., 2007	0	8	0	0	NA	NA
Yonemori et al., 2007	0	0	0	0	NA / median: 953 mL (range 200–3970 mL)	NA
Cullinane et al., 2004	15 (NA)	32 (NA)	2 (NA)	14 (NA)	NA/ mean: 565 mL (range <300 - 1,200 mL)	NA
Robles et al., 1997	11 (NA)	6 (NA)	1 (NA)	4 (NA)	mean: 696 mL (range 250 - 1500) / mean: 817 mL (range: 250–1,700)	mean: 1041 mL (range 400 - 1,700)
Kabukcu et al., 2004	12 (NA)	37 (NA)	1 (NA)	0	NA / mean: 1450 ± 280 mL	NA

Abbreviations: NA, information not available

Table S3. Most prevalent primary cancer sites/neoplasms in patients presenting with cancer-associated pericardial effusions.

First Author, Year	Most common primary cancer sites/neoplasms (No of patients)
Lobo et al., 2020	Breast (16), Lung (13), Gastrointestinal (3)
Lopez et al., 1983	Lung (8), Hematolymphoid (2)
Razek & Samir, 2019	Lung (5), Hematolymphoid (4), Breast (4), Thymoma (2), Pleural Mesothelioma (2)
Patel et al., 2013	Lung (33), Breast (17), Hematolymphoid (17), Gastrointestinal (9; 3 gastric, 2 colon, 2 pancreatic, 1 anal, 1 esophageal), Gynecological (6; 4 ovarian, 1 cervical, 1 uterine)
Sarigul et al., 1999	Breast (16), Lung (14), Hematolymphoid (6), Seminoma (2)
Wilkes et al., 1995	Lung (38), Breast (29), Hematolymphoid (33), Sarcoma (5), Gastrointestinal (7; 3 esophageal, 3 gastric, 1 colon), Gynecological (2; 1 ovarian, 1 cervical), Renal (2), Bladder (2), Melanoma (3), Germ cell (2)
Edoute et al., 1992	Lung (22), Breast (19)
Campbell et al., 1992	Breast (8), Lung (7), Gastrointestinal (3; esophageal), Head & Neck (2), Hematolymphoid (2), Gynecological (2; 1 endometrial, 1 ovarian)
Rodriguez et al., 2020	Lung (11), Breast (7), Gastrointestinal (4; 2 colon, 2 stomach), Hematolymphoid (4)
Dermawan & Policarpio-Nicolas, 2020	Lung (55), Breast (17), Gynecological (9; 8 tubo-ovarian, 1 endometrial), Hematolymphoid (12), GI (8; 6 stomach,1 esophagus, 1 colorectal), Head & Neck (2), Mesothelium (2)
Volk et al., 2019	Adenocarcinoma (11), Small Cell Carcinoma (3)
Lekhakul et al., 2018	Carcinoma and Sarcoma (141), Hematolymphoid (30)
Strobbe et al., 2017	Lung (36), Breast (12), Pleural Mesothelioma (4), Gynecological (5; 4 ovary, 1 cervical), Gastrointestinal (4; 2 esophageal, 1 gastric, 1 rectal); Hematolymphoid (2)
Gecmen et al., 2017	Lung (24), Mesothelioma (4), Gastrointestinal (3; 1 gastric, 2 esophageal), Hematopoietic (2)
Saab et al., 2016	Lung (20), Breast (12), Gastrointestinal (7), Gynecological (2), Hematolymphoid (3)
He et al., 2017	Lung (58), Breast (4), Hematologic (4), Thymoma (2), Gastrointestinal (2; esophagus), Gynecological (2; 1 ovarian, 1 cervical)
Rossi et al., 2015	Lung (9), Gynecological (4; ovarian)
Zhu et al., 2015	Lung (45), Breast (18), GI (9; 7 esophageal, 2 rectal), Gynecological (5; ovarian), Hematolymphoid (12), Mesothelioma (4)
Jeon et al., 2014	Lung (36), Breast (6), Gastrointestinal (5), Hematolymphoid (4)
Dragoescu & Liu, 2013	Lung (18), Breast (9)
Gupta et al., 2012	Breast (2), Hematolymphoid (4), Mesothelioma (2)
Mirhosseini et al.,	Lung (36), Hematolymphoid (9), Breast (7), Gynecological (4; ovarian), Bladder (3),

2012	Gastrointestinal (5; 3 colorectal, 2 stomach)
Wagner et al., 2010	Lung (78), Breast (35), Hematolymphoid (17), Gastrointestinal (13)
Maisch et al., 2010	Lung (22), Breast (8), Gastrointestinal (3; 1 esophageal, 1 colon), Hematolymphoid (2)
Hyun Kil et al., 2007	Lung (32), Breast (8), Hematolymphoid (6), Gastrointestinal (5; stomach), Gynecological (3; cervical)
Neragi-Miandoab et al., 2008	Lung (32), Breast (8), Gastrointestinal (7; 5 esophageal, 2 colon), Hematolymphoid (8)
Yonemori et al., 2007	Lung (34), Breast (13), GI (8; 6 esophageal, 1 gastric, 1 colorectal), Gynecological (2)
Gornik et al., 2005	Lung (33), Breast (16), Hematoymphoid (13), GI (9; 5 esophagus, 4 colorectal), Sarcoma (5)
Cullinane et al., 2004	Lung (15), Breast (22), Hematolymphoid (17)
García-Riego et al., 2001	Lung (39), Breast (10), Gastrointestinal (3; 2 esophageal, 1 gastric), Hematolymphoid (7)
Gupta et al., 2000	Lung (12), Breast (4), Hematolymphoid (2)
Robles et al., 1997	Breast (4), Lung (2)
Malamou-Mitsi et al., 1995	Lung (6), Epidermoid carcinoma (2), Undifferentiated (2)
Bardales et al., 1996	Lung (22), Breast (10), Hematopoietic (2)
Zipf & Johnston, 1972	Lung (3), Breast (5), Gastrointestinal (3; 2 colon; 1 gastric)
Liso et al., 2019	Lung (21), Breast (5)
Parsons & Jarzembowski, 2016	Hematolymphoid (1), Rhabdomyosarcoma (2)
Medary et al., 1996	ALL (3), AML (1), HL (1), B-cell Lymphoma (1), Medulloblastoma (1), Rhabdomyosarcoma (1), Desmoplastic sarcoma (1)

Table S4. Most prevalent primary cancer sites/neoplasms in patients presenting with malignant pericardial effusions diagnosed with cytology and/or biopsy.

Study	Lung (total, includes small cell carcinoma)	Small cell lung carcinoma	Breast	Gastrointestinal tract	Gynecological	Hematolymphoid	Others/ Unknown
**Lobo**	13		16	3	1		3
**Lopez**	8	0				2	2
**Razek**	5	0	4			4	4
**Wilkes**	24	0	24			9	13*
**Campbell**	3		4	2	1		1
**Rodriguez**	11	2	7	4	1	4	2
**Dermawan**	55	0	17	8	9	12	7
**Strobbe**	36	3	12	4	5	2	9
**Gecmen**	24	0	1	3	1	2	13
**Saab**	20	1	12	7	2	3	3
**Rossi**	9		1		4	1	21
**Zhu**	45	1	18	9	5	12	56
**Dragoescu**	18	1	9		1	1	2
**Gupta, 2012**			2			4	4
**Maisch**	22		8	3		2	7
**Miandoab**	32		8	7	1	8	7
**Yonemori**	34	2	13	8	2		3
**Garcia-Riego**	39	0	10	3		7	9
**Gupta, 2000**	12	3	4	1		2	3
**Robles**	1		2			1	
**Bardales**	22		10	1	1	2	5
**Zipf**	3		5	3		1	3

***in this study, gastrointestinal and gynecological cancer cases are included in the others/unknown category**

Table S5. Most prevalent primary cancer sites/neoplasms in patients presenting with malignant pericardial effusions diagnosed exclusively with cytology.

Study	Lung (total)	Small cell lung carcinoma	Breast	Gastrointestinal tract	Gynecological	Hematolymphoid	Others/ Unknown
**Lobo**	13		16	3	1		3
**Lopez**	7	0				2	2
**Campbell**	3		4	2	1		1
**Rodriguez**	11	2	7	4	1	4	2
**Dermawan**	55	0	17	8	9	12	7
**Strobbe**	36	3	12	4	5	2	9
**Gecmen**	24	0	1	3	1	2	13
**Saab**	20	1	12	7	2	3	3
**Rossi**	9		1		4	1	21
**Zhu**	45	1	18	9	5	12	56
**Dragoescu**	18	1	9		1	1	2
**Gupta, 2012**			2			4	4
**Yonemori**	34	2	13	8	2		3
**Garcia-Riego**	39	0	10	3		7	9
**Gupta, 2000**	12	3	4	1		2	3
**Robles**	1		2			1	
**Zipf**	3		5	3		1	3
